# Impact of dietary patterns on body weight in girls with central precocious puberty treated with leuprolide during the COVID-19 pandemic

**DOI:** 10.3389/fped.2025.1650247

**Published:** 2025-08-26

**Authors:** Fabiana Neves Figueiredo, Aline de Piano Ganen, Carolina Costa Figueiredo, Nara Michelle de Araújo Evangelista, Vânia de Fátima Tonetto Fernandes, Luciana de Aguiar Pacheco, Tatiana Kato, Guido de Paula Colares Neto

**Affiliations:** ^1^ Nutrition Department, Centro Universitário São Camilo, São Paulo, Brazil; ^2^ Pediatric Endocrinology Department, Hospital Infantil Darcy Vargas, São Paulo, Brazil

**Keywords:** dietary pattern, nutritional status, COVID-19, precocious puberty, leuprolide

## Abstract

**Objective:**

To evaluate the nutritional status and dietary patterns of girls with central precocious puberty (CPP) undergoing treatment with leuprolide acetate during the COVID-19 pandemic.

**Methods:**

A cross-sectional study was conducted with 59 girls, aged 5–13 years, diagnosed with CPP and treated with leuprolide acetate. Between May and December 2021, a food frequency questionnaire (FFQ) and a 24-hour dietary recall (24-h DR) were administered. The Fornés score was used to quantify consumption markers of healthy (beans, fruits, vegetables, and greens) and unhealthy (hamburgers, sugar-sweetened beverages, ultra-processed foods, and sweets) foods. Higher scores reflected greater intake of the respective food groups.

**Results:**

Of the participants, 31 (52.5%) showed a reduction in their BMI Z-score (mean change: −0.32 ± 0.26), while 28 (47.5%) had an increase (mean change: 0.25 ± 0.22). Girls who reduced their BMI Z-score had significantly higher Fornés scores for healthy food markers (*p* = 0.02), particularly for fresh fruits (*p* = 0.04) and greens and vegetables (*p* = 0.01). A negative correlation was found between BMI Z-score variation and the Fornés score for healthy food markers (*p* = 0.02), including fruits (*p* = 0.02) and greens and vegetables (*p* = 0.02). No significant associations were observed between BMI Z-score variation and age at treatment onset (*p* = 0.22) or treatment duration (*p* = 0.43).

**Conclusion:**

In girls with CPP treated with leuprolide acetate, greater consumption of healthy foods—especially fruits and vegetables—was associated with decreased BMI Z-scores. These findings underscore the role of dietary quality in energy balance and support nutritional monitoring during GnRHa therapy, particularly under lifestyle-altering conditions such as the COVID-19 pandemic.

## 1 Introduction

Central precocious puberty (CPP) is characterized by the early activation of the hypothalamic-pituitary-gonadal (HPG) axis, leading to pubertal development in girls younger than eight years of age (1, 2). The incidence of CPP is estimated to range from 1 in 5,000–1 in 10,000 children, occurring predominantly in girls. An increase in CPP cases was observed during the COVID-19 pandemic, a period marked by significant changes in dietary habits and a rise in obesity prevalence (3, 4).

Nutritional status is closely linked to the timing of pubertal onset. Activation of the HPG axis is associated with hormonal and metabolic changes induced by weight gain, which may lead to increased ovarian hormone production and earlier breast development. Thus, elevated body mass index (BMI) and overweight in girls may contribute to the higher prevalence of CPP (5–7).

In patients with CPP, treatment with leuprolide acetate—a gonadotropin-releasing hormone analog (GnRHa)—suppresses the production of sex hormones. This therapeutic approach delays skeletal maturation, promotes adequate linear growth, and reduces the risk of compromised adult height (3).

Although previous studies have reported an association between GnRHa treatment and increased BMI in girls with CPP, the literature remains unclear about the influence of dietary quality and quantity on anthropometric changes during this treatment, particularly in the context of lifestyle alterations brought on by the COVID-19 pandemic (8–13).

Therefore, the present study aims to assess the nutritional status and dietary patterns of girls with CPP undergoing treatment with leuprolide acetate during the COVID-19 pandemic.

## 2 Materials and methods

This study was submitted to the Research Ethics Committee of Centro Universitário São Camilo and approved under Certificate of Presentation for Ethical Consideration number 44087521.6.0000.0062 and opinion number 4.664.1111.

Participants were selected from pediatric endocrinology clinics at Hospital Infantil Darcy Vargas and the Vila Constância Specialty Outpatient Clinic in São Paulo, Brazil. Among 64 female patients aged 5–13 years, 59 met the inclusion criteria based on a diagnosis of CPP, defined as the presence of thelarche before eight years of age, accelerated growth velocity for age and sex, baseline luteinizing hormone (LH) levels above 0.3 mIU/ml, LH levels above 5 mIU/ml two hours after leuprolide acetate administration, and bone age advancement exceeding two standard deviations. All included patients were undergoing regular treatment with 11.25 mg of leuprolide acetate every 84 days for at least three months, without missed or delayed doses. Exclusion criteria included the use of other medications (glucocorticoids, anticonvulsants, antidepressants, antipsychotics) and the presence of thyroid disorders.

Data collection occurred between May and December 2021. Prior to participation, informed assent and consent were obtained from the patients and their guardians. After enrollment, data were collected from the first and last medical consultations during GnRHa treatment, and study questionnaires were administered to patients and caregivers.

Medical records included age, pubertal staging (Marshall and Tanner criteria), age at treatment onset, treatment duration, and leuprolide dosage. Anthropometric data—weight (kg), height (cm), and BMI—were collected. Height and BMI Z-scores were calculated using WHO reference standards. Nutritional status was classified by BMI-for-age-and-sex Z-scores as underweight (Z < –2), normal (–2 ≤ Z ≤ + 1), overweight (+1 < Z ≤ + 2), obesity (+2 < Z ≤ + 3), and severe obesity (Z > + 3) (14).

Food consumption was assessed using a Food Frequency Questionnaire (FFQ) and a 24 h dietary recall (24 hDR). Healthy food consumption markers (beans, fresh fruits, greens, and vegetables) and unhealthy markers (hamburgers, sweetened beverages, ultra-processed foods, and sweets) were classified according to the guidelines of the Brazilian Ministry of Health (15).

Quantitative analysis of dietary intake was conducted using the Fornés scoring system, a validated tool for assessing dietary patterns in pediatric populations, including Brazilian children and adolescents. In addition to evaluating eating behaviors, this system facilitates statistical analyses of diet quality and its associations with dietary habits (16–18).

In this method, foods reported in the FFQ and 24hDR are categorized into seven frequency ranges: f1—not consumed; f2—consumed daily; f3—once a week; f4—two to four times a week; f5—five to six times a week; f6—one to three times a month; and f7—rarely consumed (16). Each frequency category (fi) is assigned a score (Sf) based on estimated annual consumption. Daily consumption (f2) corresponds to a score of S = 1, while other categories are calculated using the formula: Sn = (1/365) × [(a + b)/2], where *a* and *b* represent the number of days in the respective frequency range (16).

For each participant, total scores were calculated separately for healthy and unhealthy food markers by summing the individual scores for the corresponding food groups. Higher scores reflect greater consumption within a given category, whereas lower scores indicate reduced intake. This approach enables a comprehensive evaluation of overall diet quality (16).

Descriptive statistics were used to summarize the data. Categorical variables were expressed as absolute and relative frequencies. Quantitative variables were presented as mean ± standard deviation (SD) for normally distributed data and median [minimum; maximum] for non-normally distributed data. Normality was assessed using the Kolmogorov–Smirnov test. Between-group comparisons were performed using the unpaired Student's *t*-test for parametric data and the Mann–Whitney *U* test for non-parametric data. Correlations between variables were analyzed using Pearson's test for normally distributed data and Spearman's test for non-normally data. A *p*-value < 0.05 was considered statistically significant. All statistical analyses were performed using SPSS Statistics version 27.0 (SPSS Inc., Chicago, IL, USA).

## 3 Results

The demographic and clinical characteristics of the study population are summarized in Table 1. The median age at the onset of thelarche was 7.0 years (4.0; 7.9), and the median age at the time of questionnaire application was 9.0 years (6.0; 13.0). At the beginning of treatment, the median age was 8.2 years (4.5; 10.7), with 76.3% of participants classified as stage III according to the Tanner and Marshall criteria. The mean duration of treatment at the time of evaluation was 16.8 ± 12.3 months. Regression or stabilization of secondary sexual characteristics was observed in 83.1% of the patients.

**Table 1:** Demographic and clinical characteristics of the study population.

Sample characteristics	Total group (*n* = 59)
Age (y)	9 (6;13)^†^
Age of thelarche (y)	7 (4;7.9)^†^
Age of treatment onset (y)	8.2 (4.5;10.7)^†^
Duration of treatment (mo)	16.8 ± 12.3*
Initial Tanner status—*n* (%)	
2	13 (22%)
3	45 (76,3%)
4	1 (1,7%)
Final Tanner status—*n* (%)	
1	7 (11,9%)
2	38 (64.4%)
3	7 (11,9%)
4	7 (11,9%)
Pubertal stabilization—*n* (%)	
Yes	49 (83.1%)
No	10 (16.9%)
Initial BMI Z- score	1.29 ± 1.21*
Initial BMI category—*n* (%)	
Underweight	1 (1.7%)
Healthy	22 (37.3%)
Overweight	21 (35.6%)
Obesity	10 (16.9%)
Severe obesity	5 (8.5%)
Final BMI Z-score	1.25 ± 1.22*
Final BMI category—*n* (%)	
Underweight	2 (3.4%)
Healthy	21 (35.6%)
Overweight	22 (37.3%)
Obesity	9 (15.3%)
Severe obesity	5 (8.5%)
BMI *Δ* Z-score	−0.05 ± 0.37*
BMI Z-score variation	
Increase (*n* = 28)	0.25 ± 0.22*
Decrease (*n* = 31)	−0.32 ± 0.26*
BMI category variation—*n* (%)	
Increase	8 (13.6%)
Maintenance	41 (69.5%)
Decrease	10 (16.9%)

*n*, number of subjects; %, percentage; y, years; mo, months; BMI, body mass index; *Δ*, difference between initial and final BMI; Values are expressed as the mean ± standard deviation *for parametric data or median [minimum; maximum] **^†^**for non-parametric data.

A total of 57 patients (96.6%) had height classified as either adequate (78%) or tall (18.6%) for their age and sex, with a mean height Z-score of 0.84 ± 1.35. Interestingly, 30 of these children (50.8%) exceeded their predicted familial height range.

At the start of treatment, 36 patients (61%) were classified as overweight or obese—a proportion that remained unchanged by the end of the treatment period. Throughout the study, 31 patients (52.5%) showed a reduction in their BMI Z-score (mean change: −0.32 ± 0.26), while 28 patients (47.5%) experienced an increase (mean change: 0.25 ± 0.22). According to WHO classification parameters, 41 patients (69.5%) maintained their BMI category despite fluctuations in BMI Z-score.

No significant correlations were found between changes in BMI Z-score and either age at treatment onset (*p* = 0.22) or treatment duration (*p* = 0.43). Additionally, there were no significant differences between the groups with increased and decreased BMI Z-scores in terms of age at treatment initiation [8.3 (5.6; 10.7) vs. 8.1 (4.5; 10.0) years; *p* = 0.23], treatment duration (17.8 ± 12.8 vs. 15.6 ± 12.0 months; *p* = 0.50) or response to treatment (75% vs. 90%; *p* = 0.70).

Patients who experienced a reduction in their BMI Z-score during treatment showed significantly higher Fornés scores for healthy food consumption markers (*p* = 0.02), including fresh fruits (*p* = 0.04) and greens and vegetables (*p* = 0.01), compared to those whose BMI Z-score increased. Moreover, this group also presented lower Fornés scores for sweetened beverages, instant noodles, snacks, and cookies—Table 2.

**Table 2:** Fornés score comparison between girls with central precocious puberty treated with leuprolide, showing whether BMI Z-score increased or decreased during the COVID-19 pandemic.

Fornés score	BMI Z-score		*p*-value
	Decrease	Increase	
	(*n* = 31)	(*n* = 28)	
Healthy food consumption markers	0.705 ± 0.207	0.574 ± 0.203	0.02**
Beans	1.00 (0.0;1.00)	1.00 (0.0;1.00)	0.79
Fresh fruits	1.00 (0.0;1.00)	0.40 (0.0;1.00)	0.04**
Greens and vegetables	1.00 (0.40;1.00)	0.40 (0.13;1.00)	0.01**
Non- healthy food consumption markers	0.378 ± 0.176	0.396 ± 0.136	0.51
Hamburgers and sausages	0.13 (0.07;1.00)	0.130 (0.02;0.70)	0.79
Sweetened beverages	0.40 (0.07;1.00)	1.00 (0.13;1.00)	0.09
Instant noodles,snacks and cookies	0.13 (0.00;1.00)	0.40 (0.07;1.00)	0.10
Sweets and treats	0.40 (0.02;1.00)	0.13 (0.02;0.40)	0.31

*n*, number of subjects; BMI, body mass index; Values are expressed as the mean ± standard deviation for parametric data or median (minimum; maximum) for non-parametric data; **p ≤ 0.05 was considered statistically significant.

A negative correlation was observed between BMI Z-score variation and the Fornés score for healthy food consumption markers (r = −0.296; *p* = 0.02), particularly for fresh fruits (r = −0.288; *p* = 0.02) and greens and vegetables (r = −0.296; *p* = 0.02). In contrast, no significant correlations were found between BMI Z-score variation and the Fornés score for unhealthy food consumption markers—Table 3.

**Table 3:** Correlations between fornés scores for food consumption markers and BMI Z-score variation in girls with central precocious puberty treated with leuprolide during the COVID-19 pandemic.

Fornés score	BMI *Δ* Z-score	*p*-value
	R	
Healthy food consumption markers	−0.296*	0.02**
Beans	−0.039^†^	0.77
Fresh fruits	−0.288^†^	0.02**
Greens and vegetables	−0.288^†^	0.02**
Non- healthy food consumption markers	−0.012*	0.92
Hamburgers and sausages	−0.134^†^	0.31
Sweetened beverages	0.134^†^	0.31
Instant noodles,snacks and cookies	0.165^†^	0.21
Sweets and treats	−0.071^†^	0.59

Correlations were performed using Pearson's test *for parametric, and Spearman's test **^†^**for non-parametric data; **p ≤ 0.05 was considered statistically significant.

Additionally, no significant differences were found between the healthy weight and overweight/obese groups in Fornés scores for either healthy (*p* = 0.24) or unhealthy (*p* = 0.44) food consumption markers.

## 4 Discussion

The initiation of treatment in the studied patients occurred later relative to the age of thelarche, with 76.3% already in Tanner stage 3 at the onset of leuprolide therapy. Despite this, 83.1% of the girls showed regression or stabilization of secondary sexual characteristics during treatment, indicating a satisfactory response to the medication and dosage regimen. These findings are consistent with previous results reported by Lee et al. and Klein et al. (19, 20). Moreover, no difference in treatment response was observed between the groups with increased vs. decreased BMI Z-scores.

Among the girls evaluated, 50.8% had a height exceeding their predicted familial growth range, a common finding in CPP. Elevated estrogen levels accelerate linear growth and bone maturation, which may ultimately result in compromised final adult height. Therefore, height monitoring plays a key role in both the diagnosis of CPP and the follow-up of GnRHa therapy, as leuprolide suppresses estrogen production and helps preserve height potential (21, 22).

Previous studies identified a correlation between BMI increase during GnRHa therapy and girls with CPP, particularly among those already overweight or obese. These findings suggest that suppression of the HPG axis by GnRHa may directly influence metabolic processes in this subgroup, thereby contributing to further increases in BMI. Specifically, the reduction of endogenous estrogen caused by treatment appears to lower basal growth rates and reduce energy expenditure. When dietary intake remains unchanged, this decrease in energy expenditure can result in additional weight gain and fat accumulation. Despite these mechanisms, 69.5% of the girls in our sample maintained their BMI category throughout the treatment period. Furthermore, no significant correlation was observed between variation in BMI Z-score and either age at treatment initiation or treatment duration (8, 9, 23–25).

Nevertheless, 61% of the girls in this study were classified as overweight or obese at the onset of treatment. Currently, the high prevalence of precocious puberty has been linked to several factors, including excess body weight and obesity, exposure to endocrine-disrupting chemicals, stressful environments, diet and adverse psychosocial conditions, especially in girls (26–29). These associations became particularly evident during the COVID-19 pandemic (2019–2022), a period marked by emergency containment measures adopted globally, including in Brazil. Social isolation, restricted mobility, and school closures contributed to changes in dietary habits, like fast food and packaged snacks, reduced physical activity, and increased sedentary behavior, ultimately leading to a rise in obesity rates (4, 30, 31).

Elevated BMI has been consistently linked to earlier pubertal onset (8). Diets high in animal protein and saturated fats, alongside low fiber intake, are also associated with premature activation of the HPG axis (32–34). This early activation is associated with elevated levels of leptin, insulin, and IGF-1. Diet-related changes in gut microbiota and inflammation may also play a role (35–37). Leptin, which is secreted in proportion to body fat, stimulates kisspeptin neurons in the hypothalamus. These neurons then trigger the release of GnRH to initiate pubertal development (38, 39). Insulin and IGF-1, often elevated in obesity, may further enhance central and peripheral HPG activation (33, 40). Additionally, the aromatization of androgens into estrogens can accelerate signs such as thelarche and menarche (33, 41, 42). Collectively, these pathways suggest that excess adiposity and poor diet contribute to both elevated BMI and earlier puberty.

During the COVID-19 pandemic, particularly following lockdown periods, an increase in the incidence of CPP and BMI gain in girls was reported (3, 7, 10–13).These observations suggest a potential causal relationship between pubertal progression and weight gain, likely driven by changes in dietary habits and lifestyle (31, 43–45). Furthermore, according to the WHO, emergency containment measures implemented to control the spread of COVID-19 may have influenced the frequency of household food purchases and dietary patterns among Brazilian families (46). In response, numerous recommendations promoting healthy eating were published during this critical period, emphasizing the role of adequate nutrition in both the prevention and management of COVID-19 infection (47–52).

In this study, the quantitative analysis of the 24hDR and FFQ using the Fornés score revealed a negative correlation between the variation in BMI Z-score and the intake of healthy food markers, including fresh fruits, greens, and vegetables (15). Additionally, the group that experienced a reduction in BMI Z-score reported lower consumption of sweetened beverages, instant noodles, snacks, and cookies compared to the group with increased BMI.

Consistent with part of our findings, Pietrobelli et al. observed an increase in fruit intake among obese Italian children during the COVID-19 pandemic; however, this was accompanied by a simultaneous rise in the consumption of processed and ultra-processed foods, such as French fries, red meat, and sugary drinks (53). Similarly, Ruiz-Roso et al. reported an increased intake of fruits, deep-fried foods, and sweets among adolescents from Spain, Italy, Brazil, Colombia, and Chile. Notably, a higher intake of greens and vegetables was observed, particularly among Brazilian girls (31). These patterns suggest that environmental factors—such as socioeconomic status, dietary trends, religious practices, and regional food traditions—may shape healthy eating behaviors. Furthermore, the WHO's guidance on promoting a healthy lifestyle during the pandemic may have contributed to an increase in the consumption of fresh fruits, greens, and vegetables (47, 50).

In our study, patients who experienced an increase in BMI tended to consume more ultra-processed foods. Eating patterns characterized by excessive and frequent intake of these products—often triggered by boredom and stress, particularly prevalent during the COVID-19 pandemic—are known to be associated with higher caloric intake and increased risk of obesity (29). According to the Brazilian Society of Pediatrics, strategies to contain the spread of COVID-19 directly impacted children's nutrition, as restrictions on mobility reduced the frequency of grocery shopping. Consequently, families may have turned to ultra-processed foods, which are easier to purchase, store, and consume due to their extended shelf life (53).

Among patients who showed a reduction in BMI Z-score, higher consumption of fruits and vegetables may have contributed to improved weight status by offsetting the caloric density and low nutritional value of ultra-processed foods.

A key strength of this study lies in the use of complementary tools to assess food consumption (24hDR, FFQ, and Fornés score), as well as the evaluation of girls with CPP undergoing GnRHa treatment during the COVID-19 pandemic—a period marked by significant lifestyle disruptions for children. Nonetheless, it is important to note that the cross-sectional design limits the ability to infer the long-term effects of the observed dietary patterns. In addition, although the 24hDR and FFQ are widely used in epidemiological research, they present limitations such as memory bias and limited accuracy in quantifying dietary intake (54).

As discussed above, among girls with CPP undergoing treatment with leuprolide acetate, higher consumption of healthy foods—particularly fruits and vegetables—was associated with a reduction in BMI Z-score. These findings underscore the important role of dietary quality in maintaining energy balance and regulating weight during GnRHa therapy, particularly during periods of lifestyle disruption, such as the COVID-19 pandemic. Clinicians must proactively incorporate tailored nutritional counseling as a standard component of CPP management. Based on these results, it is imperative to conduct robust longitudinal studies in the post-COVID period to determine if improvements in dietary habits and BMI are maintained as routines normalize with the return to in-person schooling.

## Data Availability

The original contributions presented in the study are included in the article/Supplementary Material, further inquiries can be directed to the corresponding author.
